# Functional connections between and within brain subnetworks under resting-state

**DOI:** 10.1038/s41598-020-60406-7

**Published:** 2020-02-26

**Authors:** Fabrizio Parente, Alfredo Colosimo

**Affiliations:** Dept. of Anatomy, Histology, Forensic Medicine and Orthopedics Sapienza, University of Rome, Via Borelli 50, 00100 Roma, Italy

**Keywords:** Network models, Applied mathematics

## Abstract

The focus of this paper is on the functional role of brain regions focusing on their modular architecture and individual variability. Our main assumption is that the more variable anti-correlation patterns reflect random connections, while the more conserved ones play a functional role. Within this framework, we expanded on previous results using a different database and a different methodological approach. Aiming to identify the role of specific functional connections within a global network organization which includes subnetworks, we found that the fronto-parietal module acts as the main source of anti-correlations. In addition, the pre-frontal regions (namely: frontal middle, frontal middle orbital, frontal inferior triangular) and the parietal inferior region are highly conserved and, at the same time, act as highly connected nodes, thus confirming their importance in functional modulation.

## Introduction

fMRI technology allows to explore brain regions’ activities and, in the case of spontaneous activation, reveals an organized structure with peculiar segregation and integration properties^1–3^. In addition to mutual activation among different regions (corresponding to positive correlations), another kind of interactions have been measured, namely anti-correlations (or anti-phase signal dependences)^4–8^. A major role was assigned to these interactions in the reciprocal de-activation between the Default Mode Network (DMN) and the Task Positive Network (TPN)^9–11^.

Several papers associated anti-correlation to schizophrenia^8^, working memory^6^, aging^6^, NMDA receptor^4,12^ as well as intracranial electrophysiological measurement^5,13^ and magnetoencephalographic recordings^14^. A basic methodological problem, however, was raised by Murphy^15^, underlining the role of the Global Signal Regression (GSR) method in the insertion of artifactual anti-correlations. Alternative methods were designed to face that problem^16–18^ and, more recently, evidences were obtained indicating a reproducible anti-correlation pattern between the DMN and the Dorsal Attention Network (DAN), as well as the Salience Network (SN)^19,20^.

The list of unsolved problems, however, still includes:

1. the presence of intra-module negative interactions;
2. the large inter-subject variability;
3. the specific functional role of brain regions in anti-correlated networks.

In such a context, the architecture of a negative functional brain network and its changes as a function of connection variability require systematic exploration. It is worth noting that while the individual variability’ of positive functional connection is already characterized^21^, this is not true concerning negative functional connections.

Our previous work^8^ focused on a specific condition of negative functional brain networks in which the most connected nodes have lower probability to get reciprocally connected, in contrast to the rich-club architecture^22^ of positive functional brain networks. We defined this condition in terms of a low-connection-probability range^8^.

Since the rich-club analysis only describes the behavior of the most connected nodes, we introduce a couple of new indexes to evaluate the connections of less connected nodes among each other as well as among the less and the most connected nodes. These two indexes correspond to the local and feeder connections described in van den Heuvel *et al*.^23^.

As a further step we explored the role of nodes in both positive and negative functional brain networks by functional cartography^24^. Such an analysis allows to split the nodes based on their roles within a modular architecture, namely: connector nodes (between 2 or more modules) and local nodes with or without a lower number of connections outside their own module. Notice that negative functional networks are not organized in modules, since they are lacking a clustered structure^8,15^. Conversely, positive functional networks can be efficiently sub-divided in a series of modules well overlapping to the different brain functional systems^3^. Thus, our analysis is not pointing to the modular features of negative networks as such, but to the arrangement of negative ties within and between the functional modules of the positive networks. In such a frame, most of the previous works underlined the possible functional role of negative networks in a mutual inhibition of different functional sub-networks^9–11,19,20^. However, recent evidences indicate as an alternative interpretation a possible feed-back mechanism within the same module^7^, e.g. the inhibition of the motor initiation network components in a rest condition.

In the aim to check these alternative, not necessarily conflicting, hypotheses, we focus on the arrangement of negative links within a positive functional network as a function of inter-subject variability. In our assumption, the most conserved connections could play a crucial role, being in charge of the functional de-activations among modules. The more variable anti-correlations could be associated to random events or, alternatively, to intra-module mutual de-activations emerging as transitory effects caused by contextual factors^7,20^. Finally, merging results of both analyses we could give a general picture of anti-correlations describing their local properties within the brain network organization at the global level, that is including network and subnetworks. Thus, a possible functional role of anti-correlations can be proposed and compared to the already known evidence.

## Materials and Methods

### Resting-state data collection and pre-processing

The 1000 Functional Connectomes Classic collection, Beijing Zang dataset was used (http://fcon_1000.projects.nitrc.org/indi/retro/BeijingEnhanced.html). The database includes 180 brain functional images of healthy individuals acquired in a resting-state condition at the Beijing Normal University in China with a 3.0 T Siemens scanner and the following characteristics: 240 EPI volumes; repetition time, 2000 ms; echo time, 30 ms; slices, 33; thickness, 3 mm; gap, 0.6 mm; field of view, 200 × 200 mm; resolution, 64 × 64; flip angle, 90°. The corresponding anatomical images include a T1-weighted sagittal three-dimensional magnetization prepared rapid gradient echo (MPRAGE) sequence, covering the entire brain: 128 slices, TR = 2530 ms, TE = 3.39 ms, slice thickness = 1.33 mm, flip angle 7°, inversion time = 1100 ms, FOV = 256 × 256 mm, and in-plane resolution = 256 × 192.

The following data pre-processing was carried out: the functional images were oriented to the twentieth scan, realigned and co-registered to the T1 image. Both functional and anatomical images were normalized to standard space (EPI image in Montreal Neurological Institute coordinates) using the normalization parameters of the T1 image. A spatial gaussian filter was used (4 × 4 × 4 mm), the motion parameters regressed out and a band-pass filtering in the range 0.008–0.09 Hz performed. The images were corrected by the anatomical CompCorr method^25^. SPM8 (Statistical Parametric Mapping, Wellcome Department of Cognitive Neurology, London, UK) and the Functional Connectivity Toolbox (CONN) was used on a MATLAB R2010b platform.

Finally, the Framewise Displacement method^26^ was used in order to check the movement variability in single scans. Movements above 0.2 mm were signed as bad scans, a temporal mask from the preceding to the following two scans of a bad scan was made (a sequence of 8 s), then the temporal mask was scrubbed off from the temporal sequences of BOLD acquisitions. In addition, 11 subjects having more than 60 slices in the temporal mask (120 seconds) were removed from the analysis, resulting in 169 analyzed subjects.

### Functional connectivity, small-world and efficiency analysis

The images of each analyzed subject were divided into 90 ROIs by the automatic anatomical labeling^27^ and the time-series extracted. For each subject a 90 × 90 functional connectivity matrix was estimated by a Pearson correlation coefficient between pairs of brain regions and split into two matrices, one including the positive and the other the negative values of the correlation. In both matrixes an absolute threshold method was performed spanning the 0.05 to 0.35 range of correlation values at 0.05 steps. Thus, for each subject 7 * 2 = 14 binary matrixes showing a decreasing network density were obtained.

The network efficiency is a measure of information exchanges within a network introduced by Latora and Marchiori^28^ which can be applied at both local and global scales. At the local scale the information is exchanged among each node and its neighbors; at the global scale the information exchange occurs, concurrently, across the whole network (see Supplementary Material 1).

According to Achard *et al*.^2^, a small-world range appears associated to the integration-segregation balance and characterized by values of segregation and integration higher and not-lower, respectively, than randomized networks.

Such quantities were calculated using the local efficiency as a measure of segregation, and the global efficiency as a measure of integration^28^.

We calculated the local/global efficiencies of the previous obtained binary positive networks for each threshold and for each subject. An identical procedure was followed on the corresponding random networks in order to check the statistical significance by a non-parametric Bonferroni corrected (Wilcoxon rank sum) test.

The r value found in the small-world range was used as a unique threshold for both positive and negative correlation matrices (taking the absolute value of the threshold) in order to get, after binarization, one binary network for each sign.

It must be considered that the small-world features only characterize positive functional networks and can be only used for such networks. At our knowledge, a clear procedure to select a given density of links in a negative functional network has not been proposed and in our previous work^8^ we introduced a general procedure able to threshold correlations regardless of their sign. In the present work, however, in the aim to check our previous results by a different method, we used a standard and well-known procedure for positive networks and extended the results to negative networks.

### Rich-club, feeder-club and local-club connections

In the study of the architecture of negative functional brain networks, the fraction of rich-club, local and feeder connections^23^ were respectively estimated through three indexes evaluating, for each subject, the fraction of shared links under the following conditions: among nodes with a node degree higher than a predefined value:

1 $$\phi {(k)}_{rich}=\frac{2{E}_{ > k}}{{N}_{ > k}({N}_{ > k}-1)}$$

among nodes with a node degree lower than a predefined value:

2 $$\phi {(k)}_{local}=\frac{2{E}_{ < k}}{{N}_{ < k}({N}_{ < k}-1)}$$

among nodes with node degree higher and lower than a predefined value:

3 $$\phi {(k)}_{feeder}=\frac{E-({E}_{ > k}+{E}_{ < k})}{{N}_{ > k}\,\ast \,{N}_{ < k}}$$

*where k = node degree; E = total number of network links; E*_*>k*_*, E*_*<k*_ *= number of links within nodes having node degree higher or lower than k; N*_*>k*_, *N*_*<k*_ *= number of nodes having node degree higher or lower than k.*

The first index (1) is the rich-club coefficient itself, while the other two are named local-club coefficient (2) and feeder-club coefficient (3). The whole procedure was repeated on each subject and on the corresponding randomized matrix. Since values systematically higher or lower than random were observed for the three coefficients, we defined such cases as high-connection-probability and low-connection-probability, respectively. By this approach the rich-club analysis could be extended to characterize not only the fraction of connections among highly connected nodes, but also among less connected nodes.

### Individual variability and community structure of networks

The individual variability of links was studied over the binary positive and negative adjacency matrices averaged over all subjects. Due to the binary structure, the averaged values correspond to the fraction of links present in all subjects. In other words, in the two obtained matrices a link’s weight corresponds to the frequency of that link in the sample. The matrices were filtered by a series of increasing thresholds from 0.05 to 1, so that a matrix with threshold = 1 contains the most conserved links, namely links shared by 100% of the subjects.

A network is endowed with a community structure if the nodes can be grouped into internally densely connected sets: this can be quantified by evaluating a Q index (see Supplementary Material 1). We carried out a modularity^29^ study on the positive connection variability matrix using the maximal threshold values compatible with a connected network, namely in the absence of isolated nodes. Under that condition, the Q index and the number of modules were derived. The anatomical location of nodes as visualized over standard brain pictures is reported in Supplementary Material 2. The variability of negative functional connections between and within modules was explored according to a Functional Cartography analysis^24^, which characterizes the nodes by two indexes: the within-module degree (z) and the participant coefficient (P). z and P measure, respectively, the node degree of nodes within their own module (intra-module connectivity) and among modules (inter-modules connectivity).

### Analytical approach

The scripts to calculate the previously described network indexes were taken by the Matlab Brain Connectivity Toolbox (BCT)^30^, except for the feeder- and local-club coefficient in which an original home-made script was used (see Supplementary Material 3).

To produce random networks the Maslov and Sneppen method^31^ was used, in which a network is randomized preserving the degree distribution.

In all cases, the statistical significance of the observed events was verified using a Bonferroni corrected, Wilcoxon test.

Supplementary Material 1 contains the definition of the metrics and the analytical details of the calculation.

The anatomical location of nodes as visualized over standard brain pictures is reported in Supplementary Material 2. Supplementary Material 3 contains a global flow-chart of the analytical strategy followed in the paper as well as a small toy-network showing the methods used in the study of Adjacency Matrixes.

### Ethical approval

All procedures performed in studies involving human participants were in accordance with the ethical standards of the institutional and/or national research committee and with the 1964 Helsinki declaration and its later amendments or comparable ethical standards.

### Informed consent

Informed consent was obtained from all individual participants included in the study.

## Results

### Efficiency analysis

**Figure 1:**
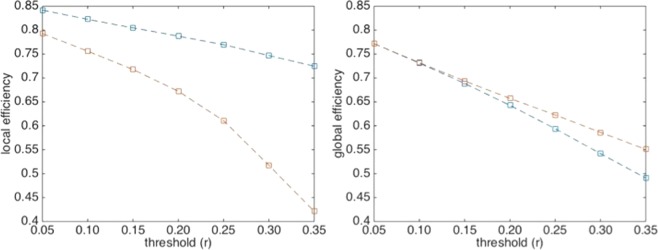
Efficiency analysis of positive correlation matrixes. Left and right panels contain, respectively, local and global efficiency values as a function of the threshold used to filter Pearson correlations (see data processing). Blue and red lines refer to actual and randomized data, respectively.

The analysis of positive correlations, reported in Fig. 1, shows a local efficiency higher than that of the corresponding randomized data at any threshold (p-value <0.001), while the global efficiency remains close to random up to r = 0.15. This value is used as a threshold in subsequent analyses for both positive and negative functional networks. Table 1 shows clear differences between positive and negative functional brain networks in the whole set of indexes. According to our previous results^8^, assortativity shows positive and negative value in positive and negative functional brain networks, respectively. At the same time negative networks appear less connected as compared to the positive ones. Another noteworthy result concerns the local efficiency in the negative network which is significantly lower than the random network (p-value <0.0001), thus confirming the absence of clusters in the network^8,15^.

**Table 1:** Positive and negative functional brain networks’ parameters at threshold | r | = 0.15. Mean values ± S.D. are provided for all examined subjects. (Details in Supplementary Material 1).

	Functional brain networks	
	Positive	Negative
Assortativity	0.24 ± 0.11	−0.34 ± 0.1
Network Density	0.39 ± 0.07	0.17 ± 0.05
Local efficiency	0.80 ± 0.02	0.09 ± 0.06
Global efficiency	0.63 ± 0.01	0.62 ± 0.1
Node degree	34.5 ± 5.8	15.1 ± 4.2

### Extended rich-club analysis

A rich-club analysis was carried out on the positive and negative matrices in the integration-segregation interval (threshold = 0.15) and the associated coefficients were calculated for each node degree and for each subject, as well as for the corresponding randomized matrixes.

**Figure 2:**
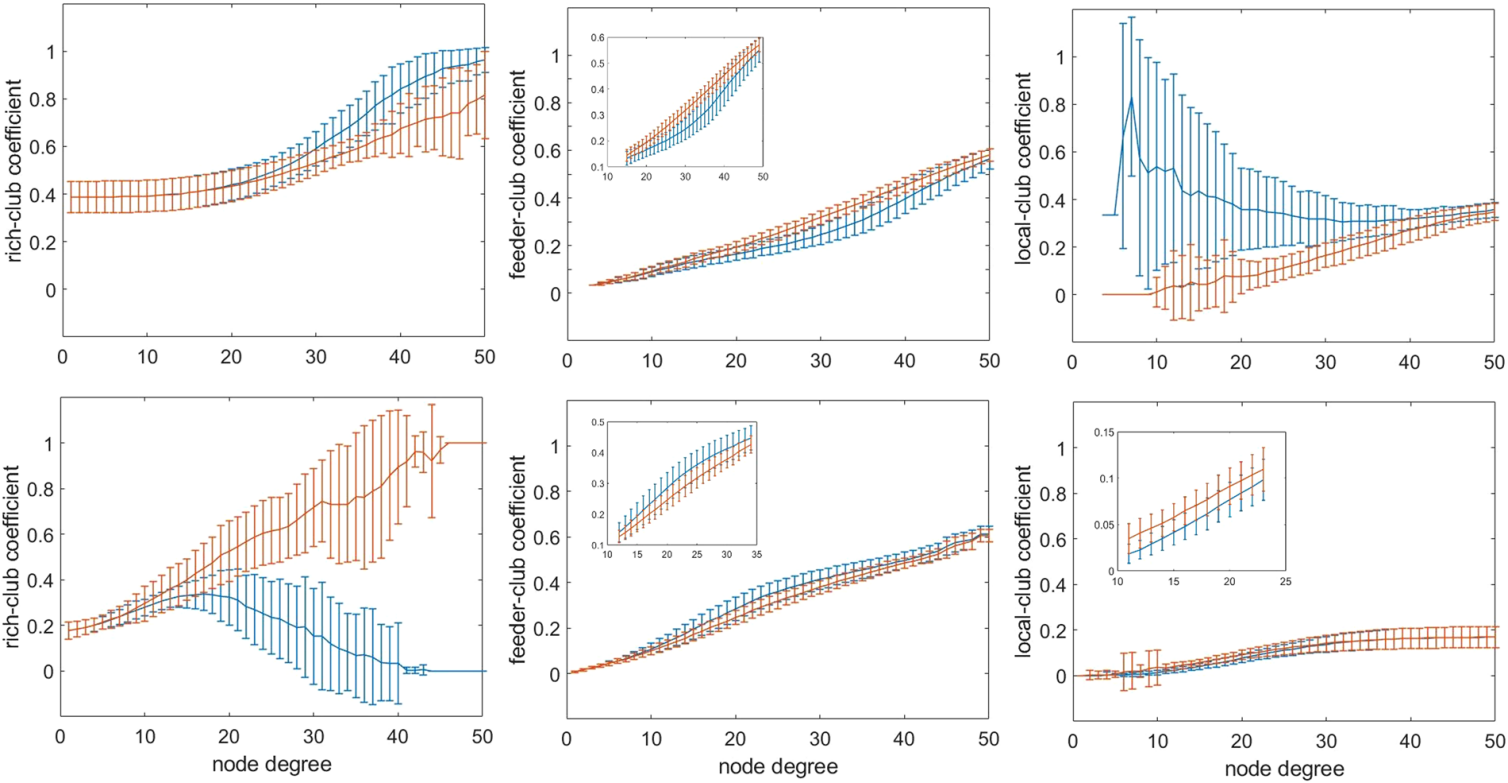
Rich-club analysis under different node degree values. Top row: positive functional brain networks; bottom row: negative functional brain networks. The inserts show an enlargement of the critical regions. Blue and red lines indicate actual and randomized networks, respectively.

As reported in Fig. 2 (left column) the rich-club coefficient has high- and low-connection-probability intervals spanning approximately the 26–50 and 13–45 node degree range for positive and negative matrixes, respectively. More interesting is the behavior of the feeder-club coefficient (middle column), which shows low- and high-connection-probability intervals in the middle node degree range: 15–49 and 12–34, respectively. Finally, the local-club coefficient (right column) of positive matrixes has another high-connection-probability interval in the less connected nodes, while negative matrixes have a small decrease trend of connection-probability for the less connected nodes (positive: 10–44 node degree; negative: 6–23 node degree).

**Figure 3:**
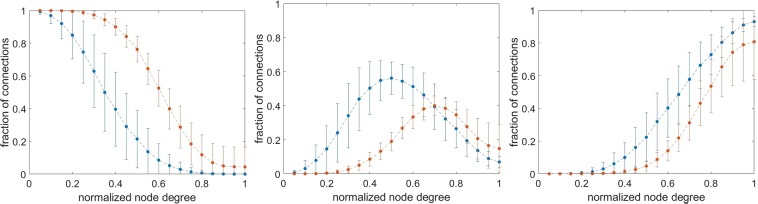
Variability of % connections as a function of normalized node degree. Fraction of rich, feeder and local connections are reported in the left, middle and right panels, respectively. Red and blue lines refer to positive and negative networks, respectively. The node degree was normalized to the maximum value for each subject. As for the feeder connections, notice the lower peak of the positive functional brain networks as compared to the negative ones. The fraction of connection is calculated taking into consideration the total number of links in the matrix, namely summing the rich, feeder and local connections at the given normalized node degree.

For the sake of comparison, we considered the fraction of connections as a function of the node degree normalized for the total number of links for each subject. The following differences unrelated to connection density emerge (Fig. 3): positive matrices reach maximal values of feeder connections at 0.69 normalized (37 not normalized) node degree, corresponding to fractions of rich, feeder and local connections of 0.29 ± 0.13; 0.40 ± 0.06; 0.31 ± 0.11, respectively.

Negative matrices reach maximal values of feeder connections at 0.51 normalized (19 not normalized) node degree, corresponding to fractions of rich, feeder and local connections of 0.22 ± 0.18; 0.56 ± 0.10; 0.22 ± 0.15, respectively.

Since the maximal values of feeder connections indicate the maximal number of connections between nodes having higher and lower node degree, we used that value to classify nodes as central and non-central nodes. Table 2 includes the central nodes of positive and negative functional brain networks with the associated node degree.

**Table 2:** Mean Node Degree values (±S.D.) of central nodes in positive and negative functional brain networks. The correlation matrixes from all subjects were binarized and thresholded at |r | = 0.15.

Positive functional brain Networks	Negative functional brain Networks
Temporal pole superior right 44.7 ± 11.2	Parietal inferior right 24.2 ± 11.0
Temporal pole superior left 43.6 ± 10.5	Frontal middle right 23.3 ± 10.6
Temporal superior right 43.2 ± 1.1	Frontal inferior opercular right 23.2 ± 10
Parahippocampus right 42.6 ± 9.8	Parietal inferior left 22.6 ± 10.3
Fusiform left 40.7 ± 11.7	Frontal inferior triangular left 21.7 ± 10.5
Fusiform right 41.2 ± 11.2	Frontal middle orbital right 21.6 ± 11.2
Temporal superior left 41.4 ± 10.7	Angular right 21.5 ± 9.3
Temporal middle right 41.4 ± 11.0	Frontal middle left 20.9 ± 10.8
Temporal middle left 41.2 ± 10.4	Supramarginal right 20.9 ± 9.1
Parahippocampus left 39.9 ± 9.5	Frontal middle orbital left 20.9 ± 10.8
Frontal medial orbital left 39.3 ± 8.4	Angular left 19.8 ± 8.6
Cingulum anterior left 39.1 ± 9.4	Cingulum posterior right 19.3 ± 8.7
Cingulum middle right 39.0 ± 9.8	Supramarginal left 19.2 ± 9.0
Frontal medial orbital right 38.8 ± 8.6	Frontal inferior triangular right 19.0 ± 9.8
Temporal pole middle left 38.7 ± 7.9	
Post-central right 38.4 ± 9.6	
Temporal pole middle right 38.2 ± 9.8	

### Modularity of functional connections

**Figure 4:**
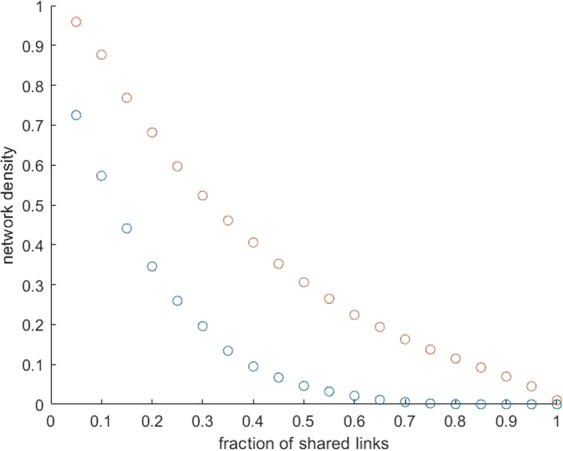
Network density as a function of the fraction of shared links. Red and blue circles refer to positive and negative functional brain networks, respectively.

Figure 4 shows that the density of both positive and negative mean matrices (derived in section 2.4) decreases with the increasing fraction of shared links. However, the density of the negative functional brain network declines to 0 at 85% shared links, while the positive one still shows 1% of network density at the maximum fraction (100%) of shared links. Choosing the optimal fraction of shared links in the assessment of the modules’ number must take into consideration that above the 75% of shared links (at positive network density = 0.15) the positive functional brain network becomes disconnected. Thus, the above value will be used as a threshold in the subsequent analyses and will produce a Q value of 0.52 as well as six modules. Two modules cover most of the fronto-temporo-parietal brain areas, including the Task-Positive Network (TPN). We called the former one fronto-parietal subnetwork and the latter one temporo-parietal subnetwork. Another module covers a sparse set of regions in the limbic brain and was called limbic subnetwork. A fourth module is formed by brain regions overlapping the Default Mode Network (DMN). Finally, the last 2 modules are formed by occipital/vision related brain regions and basal ganglia regions, respectively.

**Figure 5:**
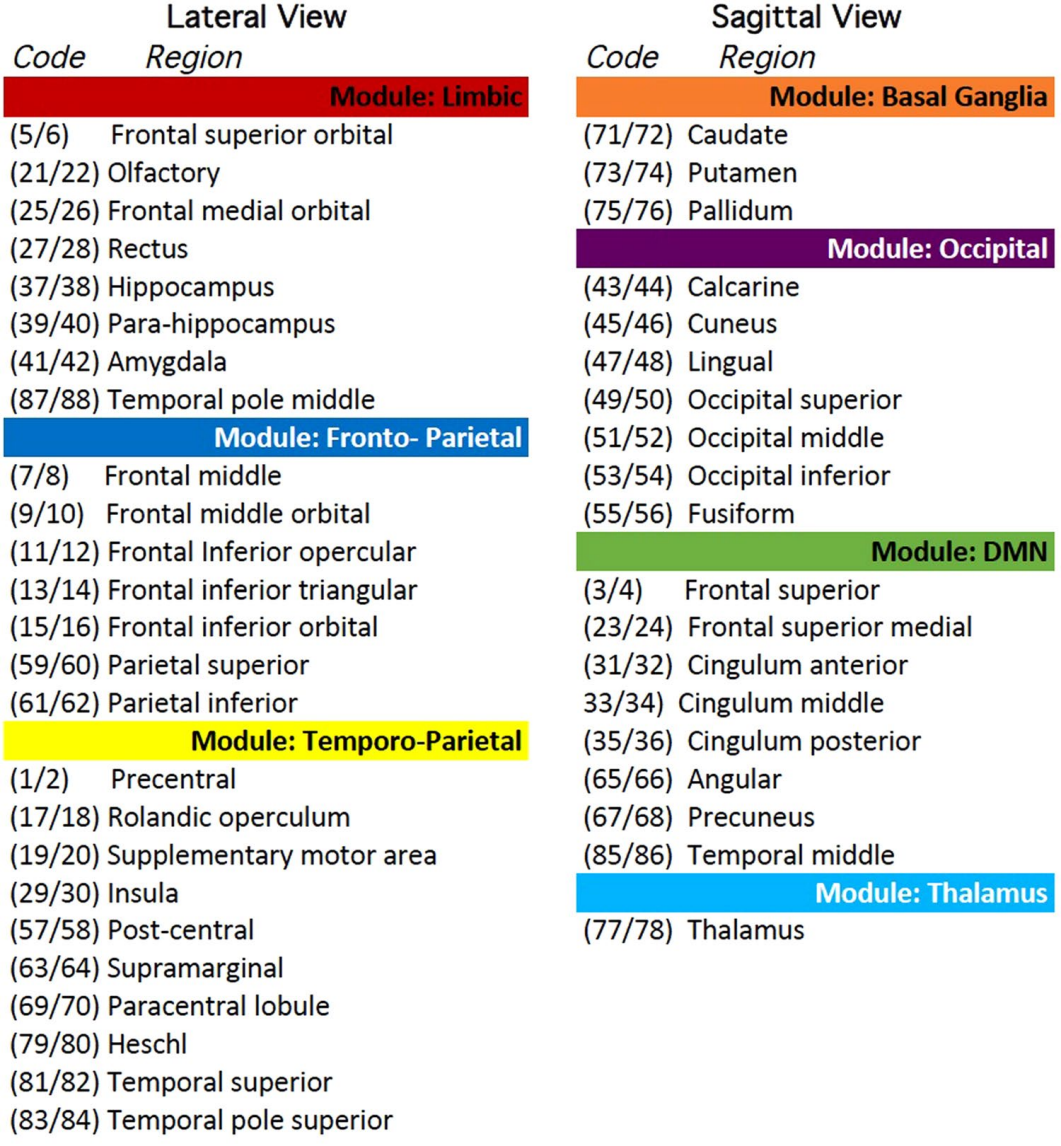
Localization of brain regions within functional modules. The numeric codes refer to the 90 ROIs in Tzourio-Mazoyer *et al*.^26^ odd and even tags refer to right and left hemisphere, respectively. For the sagittal and lateral brain views see the Supplementary Material 2.

Some regions show unexpected modular location: (1) the left and right middle cingulate cortex are split in the temporo-parietal and DMN module respectively; (2) the left and right pre-central are separated in the fronto-parietal and temporo-parietal subnetworks; (3) the thalamus is inserted in the DMN module. We studied in deep the connections of these regions in order to understand possible miss-matched results and could define that: (1) the left middle cingulate has 47% of its connections in the DMN module (that contains its contralateral part) and 53% in the fronto-temporal, then its connections are placed between the two modules almost symmetrically; (2) the left pre-central gyrus has 42% of connection on its module (fronto-parietal) but 58% in the temporo-parietal module (more appropriate for the somatic-motor function); (3) the thalamus is connected to the DMN by a single link (right thalamus − right middle cingulum). Thus, the left middle cingulate was included in the DMN module, the left pre-central gyrus in the temporo-parietal module and the thalamus in an independent module. It is worth noticing that at a stricter threshold level (0.80) the above regions are segregated in the same way. For detailed information about modules see Fig. 5. The anatomical location of brain regions is shown in the Supplementary Material 2.

### Functional cartography of brain networks

A functional cartography analysis separately performed over positive and negative networks clarified the specific role of nodes in the two conditions. As for positive networks, we used the same threshold level selected in the modularity study (75%); as for negative networks, a series of increasing threshold levels were tested in order to verify the presence of variability.

**Figure 6:**
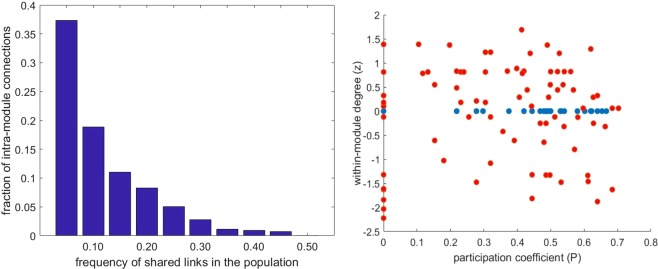
Intra- and inter-module connections of positive and negative functional brain networks. Left panel: distribution within the considered subjects of the fraction of intra-module negative connections. Right panel: Functional cartography of positive (red dots) and negative (blue dots) connections at different values of shared links (75% and 50%, respectively) within the considered subjects (See the text for details).

Concerning the negative network, Fig. 6 (left panel) shows that at increasing threshold values a decreasing fraction of within-module negative links is observed up to 50% (corresponding to 0.03 network density) and above that only negative between-modules links do exist. Figure 6 (right panel) shows the functional cartography indexes of positive and negative functional brain networks. In the case of the negative ones (blue dots) a threshold of 50% has been chosen, due to the results in the left panel: the whole set of nodes shows z = 0, indicating inter-module connections only; the participation coefficient (P) appears well distributed among nodes. Concerning positive networks (Fig. 6, red dots in the right panel), no node shows a within-module degree higher than 2, meaning that the node degree inside modules is equally distributed. The P values fluctuate from 0 to 0.7, meaning that several nodes are not connected to other modules but some nodes tend to connect to other modules through at least 50% of their links (P > 0.62) accounting for 9% of the total amount of nodes (bilateral frontal inferior orbital, left frontal superior medial, left parietal superior, left precuneus, right putamen, left temporal pole superior and left temporal middle).

**Figure 7:**
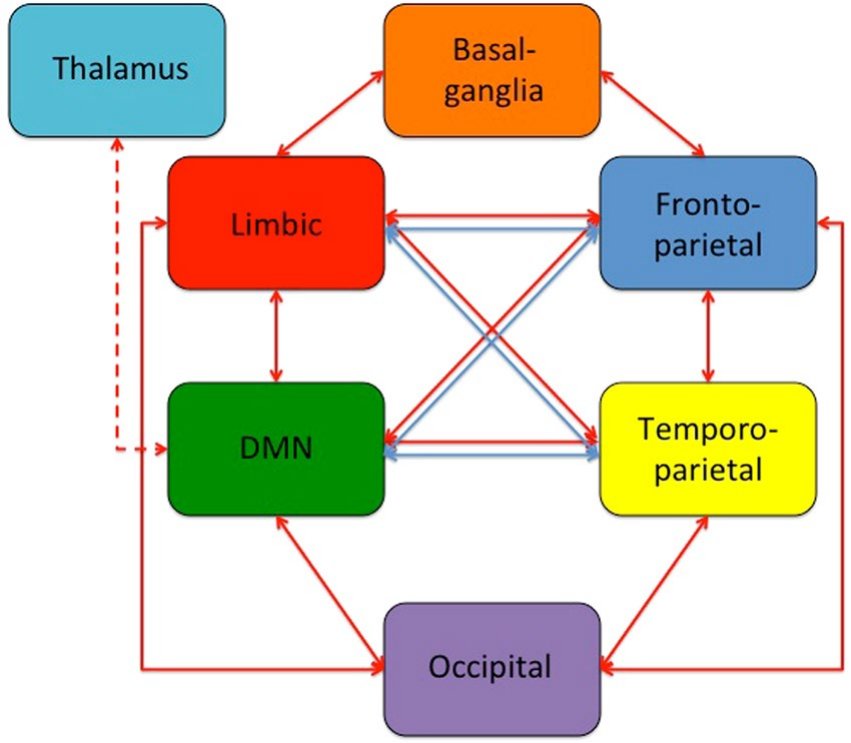
Functional connections among brain subnetworks. Red arrows: positive connections; Blue arrows: negative connections. The Thalamus is only connected to the DMN through the middle cingulate (dotted arrow). The connections refer to the 75% and 70% threshold level for positive and negative connections, respectively. Details on brain modules and regions in Fig. 5.

A further characterization of the node features, can be obtained by looking at the module-by-module connectivity, as reported in Fig. 7. As a general comment for positive functional brain networks, the basal-ganglia module is only connected with the limbic and temporo-parietal modules, while the others (including temporo-parietal and limbic modules) have at least one common link among each other. Concerning the negative functional brain network, anti-correlations are present at 50% threshold among all modules, except basal ganglia. However, at 55% threshold the anti-correlations between the fronto-parietal and the temporo-parietal modules (post-central left to bilateral frontal middle) disappear, while anticorrelations remain between the fronto-temporal module and the occipital module (up to 60% threshold), as well as between the temporo-parietal module and the occipital module.

### Connections variability of peculiar node roles

**Figure 8:**
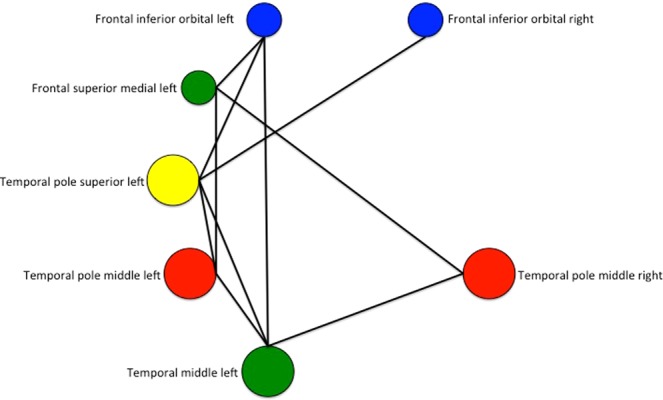
Functional connections among connector nodes in the left and right hemispheres. Central nodes are bigger. The color codes are the same used in Fig. 7.

On the basis of the previous analyses, the role of single nodes together with the variability of their connections can be explored. In Table 3 the inter-modules connections associated to the connector nodes in the positive functional brain network are listed. The most connected modules are the DMN and the limbic, via the medial part of frontal gyrus and the temporal polar regions. The TPN subnetworks also appear highly connected via the lateral frontal and temporal pole regions. It is worth to note that the only connections of the basal ganglia are between the putamen and both: the amygdala and supplementary motor area, for the limbic and the temporal-parietal way, respectively. Finally, the TPN and DMN connections go mainly through the lateral and medial frontal regions, as well as via temporo-temporal connections. Figure 8 shows that the nodes having both centrality and connectors features are placed in the temporal regions, which are included within a central-core (super-modular) structure.

**Table 3:** Inter-modules positive connections mediated by connector nodes.

*Fronto-parietal* ↔ *Temporo-parietal*	Frontal inferior orbital left - Temporal pole superior left ** Frontal inferior orbital right - Temporal pole superior left * **Frontal inferior orbital right** - Temporal pole superior right *
*Fronto-parietal* ↔ *DMN*	Frontal inferior orbital left - Frontal superior medial left * Frontal inferior orbital left - Temporal middle left * **Frontal inferior orbital right** - Frontal superior medial right * **Frontal inferior orbital right** - Temporal middle right *
*DMN* ↔ *Limbic*	**Frontal superior medial left -** Frontal medial orbital left ** **Frontal superior medial left** - Frontal medial orbital right **Frontal superior medial left - Rectus right** **Frontal superior medial left** - Temporal pole middle left * **Frontal superior medial left** - **Temporal pole middle right** Frontal superior medial right - **Temporal pole middle left** Frontal superior medial right - Temporal pole middle right ** **Temporal middle left** - Temporal pole middle left ** **Temporal middle left** - Temporal pole middle right ** Temporal middle right - **Temporal pole middle left** Temporal middle right - Temporal pole middle right **
*Limbic* ↔ *Temporo-parietal*	**Temporal pole middle left** - Temporal pole superior left ** Temporal pole middle right - **Temporal pole superior left** Temporal pole middle right - Temporal pole superior right
*DMN* ↔ *Temporo-parietal*	**Temporal middle left** - Temporal pole superior left * **Temporal middle left** - Temporal superior right
*Basal ganglia* ↔ *Temporo-parietal*	**Putamen right** - Supplementary motor area right
*Basal ganglia* ↔ *Limbic*	**Putamen right** - Amygdala left **Putamen right** - Amygdala right
*DMN* ↔ *Occipital*	**Precuneus left** - Cuneus left ** **Precuneus left** - Cuneus right **

Right: name of connected modules; left: single connection names. The data refer to 75% fraction of shared links within the considered subjects; * and ** indicate connections still present at shared links fractions higher than 90% and 95%, respectively (see Data Processing section). Connector nodes endowed with P > 0.62 are in bold.

Concerning negative connections, the most conserved anti-correlations remain those between the DMN module and the TPN modules (fronto-parietal and temporo-parietal, at 75% threshold), as well as those between the limbic module and the TPN modules (at 80% threshold, maximal value for anti-correlations). From Table 4 one can conclude that the fronto-parietal module is the main negative modulator of the brain functional system. The following regions are endowed with the higher number of negative central nodes and, at the same time, with the less variable connections: frontal middle, frontal inferior opercular, frontal inferior triangular, parietal inferior. Moreover, in the right panel of Fig. 6 a noticeable scattering of participation coefficient (P) values of negative functional brain networks appears, underlining that a single node can be characterized by a variable number of connected modules: the higher such value, the more modules are negatively connected to that node. Thus, the previous threshold value for connector nodes (P > 0.62) indicates the nodes with multi-module de-activations: frontal middle orbital right, frontal inferior opercular right, frontal inferior triangular right, parietal inferior right and supramarginal right. It can be noticed that such regions refer to the central nodes of the right hemisphere.

**Table 4:** Inter-modules connections of negative functional brain networks.

*Fronto-parietal* ↔ *Limbic*	Frontal middle right - Frontal medial orbital left * Frontal middle right - Rectus left * Frontal middle right - Hippocampus left * Frontal inferior opercular left - Frontal medial orbital right **Frontal inferior opercular right** - Frontal medial orbital left ** Frontal inferior triangular left - frontal medial orbital right **Parietal inferior right** - frontal medial orbital left ** **Parietal inferior right** - rectus left
*Fronto-parietal* ↔ *DMN*	Frontal inferior opercular left - Precuneus right * Frontal inferior triangular left - Cingulum anterior right Frontal inferior triangular left - Precuneus right * Frontal inferior orbital left - Precuneus right Parietal superior right - Frontal superior medial left **Parietal inferior right** - Frontal superior medial left Parietal superior left - Frontal superior medial right
*Temporo-parietal* ↔ *Limbic*	Insula left - Frontal medial orbital left Insula right - Frontal medial orbital left **Supramarginal right** - Frontal medial orbital left
*Temporo-parietal* ↔ *DMN*	Rolandic operculum left – Angular right Rolandic operculum right – Angular right * **Supramarginal right** – Frontal superior medial left Insula left – Angular right

Right: name of connected modules; left: single connection names. The data refer to 70% fraction of shared links within the considered subjects; * and ** indicate connections still present at shared links fractions higher than 75% and 80%, respectively (see Data Processing section). Connector nodes endowed with P > 0.62 are in bold. Frontal middle orbital right and frontal inferior triangular right although defined as connector nodes are not included in the Table since do not survive at the threshold used.

## Discussion

### Structure and variability of negative connections

In a previous work we introduced a specific method to highlight significant properties of negative connections in brain functional networks^8^ based upon a fixed threshold of connections’ density. In the present paper, we confirm those results using a different strategy and a different database. We additionally address two specific questions concerning (1) the variability of negative brain interactions, and (2) their role within functional modules. Concerning the changing role of nodes as a function of connections variability, a limit in the variability of the intra-module connections clearly appears. Nodes endowed with intra-module roles, in fact, are only found in less conserved (namely highly variable) connections, meaning that the intra-module de-activations could be functionally non-significant. On the other hand, their low density allows for an alternative hypothesis: while the most conserved connections could have an intrinsic role in the separation of functional modules, the others could play a transitory intra-module de-activation role as a function of subject-related factors^7,20^. In order to clarify this point we plan to explore whether specific phenotypes (gender, age, psychological features, clinical characterization, etc.), as well as particular task conditions, can be associated to such highly variable connections. However, from the present results it appears that a small amount of anti-correlations plays a role in the reciprocal de-activations of specific modules, in agreement with the evidence provided by several authors that the TPN and the DMN are the main subnetworks with reciprocal de-activations^9–11^.

The effective connectivity studied by Zhou *et al*.^32^ and Almagren *et al*.^33^ defines the fronto-parietal regions as main inhibitory modulators. According to our results, the corresponding overlapped regions (parietal lobes and inferior frontal gyrus) appear having higher scores in participation coefficient (P). Moreover, in Zhou *et al*.^32^ such interactions are not bidirectional indicating a directed modulation of DMN.

**Figure 9:**
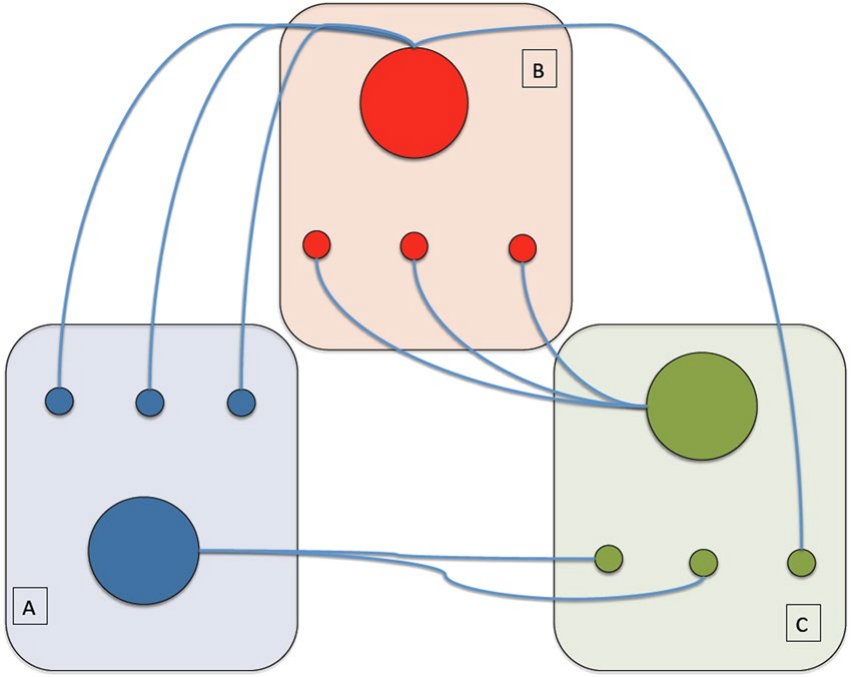
Schematic of a negative brain functional network. Blue, red and green functional modules (indicated by A, B and C) each include 4 nodes. Within the same module nodes do not interact among each other. The central (bigger) node in each module only interacts with the less connected (smaller) nodes of the other modules. Note that the red central node interacts with two different modules, which means a higher P value compared to the other two central nodes.

High value of P in negative networks indicates nodes having negative functional links with different modules. Figure 9 shows an example of an architecture concerning a node negatively interacting with different modules. Thus, a possible relation between multi-modules anti-correlated regions and causal interactions could be considered.

The rich-club, feeder-club and local-club analyses entail some interesting consideration concerning anti-correlations. The main finding is a low-probability interval in more and in less connected nodes (Fig. 2, left and right panels in the bottom row). These results are in line with the general disassortative nature of the negative functional brain network^8^. Furthermore, the feeder-connections (between the more and the less connected nodes) show a node degree interval with higher probability of connections than the corresponding random model, indicating an over-expression of this kind of link. All in all, our results helped developing a general model of a negative functional brain network where nodes belonging to the same functional module are not connected between each other, but a few central nodes (hubs) take a connection with the less connected nodes of other modules. In Fig. 9 a minimal scheme of such a model is proposed.

### Comparison of positive and negative functional brain networks

Besides confirming the basic architecture of a negative functional brain network, our results deal with positive functional brain networks too. In this case, the architecture shows very cohesive connections among nodes endowed with a low node degree, as well as among central nodes, which is in line with the assortative nature of the positive functional brain network, and different from the disassortative feature of the negative network. Moreover, the result found in the feeder-connections (Fig. 2, middle column) shows another evidence of opposite behavior of positive and negative brain functional networks, describing a lower- and higher-connection-probability in the positive and negative network, respectively. It is worth stressing that the difference between positive and negative functional brain networks is independent on network densities and might be due to specific physiological roles.

A careful exploration of nodes’ role indicated the presence of connector nodes but no intra-module hubs. In addition, the connector nodes not necessarily coincide with central nodes. This means that the rich-club description of functional brain networks does not include inter-modular connections, as in the core-structure model found by van den Heuvel *et al*.^23^, on the basis of anatomical connections estimated by Diffusion Tensor Imaging (DTI). Our results are in line with Grayson *et al*.^34^, who hypothesize the more segregated structure of the positive functional brain network as a possible reason. However, comparing the features of single nodes, we found that temporal regions (temporal pole superior, temporal middle and temporal pole middle) have both connector and central node features (Fig. 8). Since these regions also show quite conserved links (low inter-subjects’ variability), we may suggest for them a specific role in the functional balance of an integrated system. Previous works exploring anatomical connectivity found as central nodes of the brain network the fronto-temporo-parietal regions^1,22^.

From our results similar brain regions can be defined as central nodes within differently signed (positive/negative) functional brain networks. Starting from the obvious assumption that, in the lack of functional information, structural data may only provide a partial picture of the brain system, we would like to stress the importance of the functional dimension in the subtle dynamical information exchange among brain nodes. In such a context, among other things, we could confirm the fronto-parietal and the temporal regions as important communication centers showing a mutual de-activation and a co-activation role, respectively.

### Limitations of this study

A limitation deals with the recently characterized^35^ time-dependent properties of the functional connectivity: we didn’t explore that in the case of anti-correlations. Since the whole subject of anti-correlations still is on debate we privileged, at this stage, a solid characterization of their static features.

According to the same reasoning and in the issue of the task-related connectivity changes^6,8^, we focused, as a preliminary approach, looking for a minimal description of anti-correlated networks in a resting-state condition. Further studies of task-evoked activity are needed to assess the cognitive role of negative correlations.

Another relevant limitation might appear the calculation of the participation coefficient (P), as well as the within-module degree (z), which are related to the particular modular architecture used as a shaper^36^. Thus, our results are related to the modularity analysis found in our dataset. However, our modules do agree with previous reports^3^ and with the already known functional sub-networks. Furthermore, in order to avoid any confusion, we inserted in Tables 3 and 4 the information on single connections and node labels.

Last but not least, since different results can be obtained using different brain atlases^37^, we realize the interest of checking the variability of negative networks along the same line and plan to investigate the issue as soon as possible.

## Conclusion

The aim of this work is to improve the exploration of negative functional brain networks properties at both local and global levels and to propose a model to clarify their physiological role. In this frame, from the comparison between such properties and the inter-subject’s variability, some specific measures potentially useful to detect individual differences in physiological and/or pathological contexts were derived. Completely open remains the modeling the variability of functional networks at different time scales, in the aim to associate the intrinsic excitability of the system to the continuously changing and demanding environmental constraints.

## Supplementary information

Supplementary Material.
