# Supplementary material for: Functional connections between and within brain subnetworks under resting-state
Source: Sci Rep. 2020 Feb 26;10:3438. doi: 10.1038/s41598-020-60406-7 (PMC7044315; doi:10.1038/s41598-020-60406-7)
Supplement: Supplementary file 1 — Supplementary Material. [file 41598_2020_60406_MOESM1_ESM.pdf]

Functional connections between and within brain subnetworks [Supplementary Material]

Fabrizio Parente* and Alfredo Colosimo*

*Dept. of Anatomy, Histology, Forensic Medicine and Orthopaedics Sapienza. University of Rome

1 Metrics and jargon used in this paper.

Basic Notation:
N = set of all nodes in the network;
n = total number of nodes in a network;
k = node degree;
l = total number of links in a network.

- *Rich-club coefficient:*
  Networks having a relatively high rich-club coefficient are characterized by many connections between nodes of high degree [1].

$$\phi(k)_{rich} = \frac{2E_{>k}}{n_{>k}(n_{>k}-1)} \tag{1}$$

  where $E_{>k}$ = number of links within nodes $n_{>k}$, having node degree higher than k.

- *Small-world phenomenon:*
  It is generally defined in terms of segregation-integration balance. In the present paper segregation and integration were evaluated through local efficiency and global efficiency, respectively.

- *Efficiency:*
  It has been proposed to evaluate the information exchange within a network [2]. The concept can be applied at both local and global scales. *Local efficiency* = measure of the information exchange between each node and its neighbors [3]:

$$E_{loc} = 1/n \sum_{i \in N} \frac{\sum_{j,h \in N; j \neq i} a_{ij} a_{ih} [d_{jh}(N_i)]^{-1}}{k_i(k_i - 1)} \quad (2)$$

  where a_{ij} = binary value equal to 1 if a link between i and j node does exist; d_{ij} = shortest path length between nodes i and j.
  Global efficiency = measure of the information exchange across the whole network

$$E_{glo} = 1/n \sum_{i \in N} \frac{\sum_{j \in N; j \neq i} d_{ij}^{-1}}{n - 1} \quad (3)$$

- *(Dis)assortativity:*
  Pearson correlation coefficient used to evaluate up to what extent nodes associate with other nodes sharing similar or different characters. In a disassortative network high degree nodes are connected, on average, to nodes with low(er) degree and, on average, low degree nodes are connected to high(er) degree nodes.

- *Modularity:*
  A module is a set of nodes densely connected internally. If the nodes can be grouped into potentially overlapping and highly interconnected sets, the concept allows for a coarse-grained, and thus simplified, description of the network and of its community structure. The presence of community structures in networks, including non-overlapping modules, is based upon the evaluation of the Q index [4].

- *Q index:*
  Q is defined as the normalized fraction of links in a module minus the expected number in an equivalent random network [4]:

$$Q = \frac{1}{l} \sum_{i,j \in N} (a_{ij} - \frac{k_i k_j}{l}) \delta_{m_i m_j} \quad (4)$$

  where a_{ij} = binary value equal to 1 if a link between i and j node does exist; k_i and k_j = node degree of nodes i and j; m_i and m_j = module of i and j; $\delta_{m_i m_j}$ = Kronecker delta with value 1 if $m_i = m_j$, 0 otherwise.

- *Functional cartography:*
  Method able to extract information from the topology of a complex network by means of the z and P indexes [5].
  Within module node degree (z_i): measure of the intra-module connectivity, for node i, z_i spans from -∞ to $+\infty$ and nodes with z $>$ 2.5 are defined *hubs*:

$$z_i = \frac{k_i(m_i) - < k > (m_i)}{\sigma^{k(m_i)}} \tag{5}$$

  where: m_i = module containing node i; $k_i(m_i)$= intra-module node degree of node i (the number of links between i and all other nodes in m_i); $< k > (m_i)$ = average of the intra-module node degree of nodes within-module m_i; $\sigma^{k(m_i)}$= standard deviation of the intra-module node degree of nodes within-module m_i.
  Participation coefficient (P_i): measure of the inter-module connectivity, for node i, P_i spans from 0 to 1, if the connections are within their own module, or distributed in all modules, respectively:

$$P_i = 1 - \sum_{m \in M} (\frac{k_i(m)}{k_i})^2 \tag{6}$$

  where: M = set of modules; $k_i(m)$ = number of links between i and nodes in module m.

- *Null statistical models by network randomization:*
  The statistical significance of the results was checked by the straightforward method in [6] whose main advantage is the preservation, in the randomized network, of the degree distribution present in the original network.

2 Anatomical location of brain regions and modules.

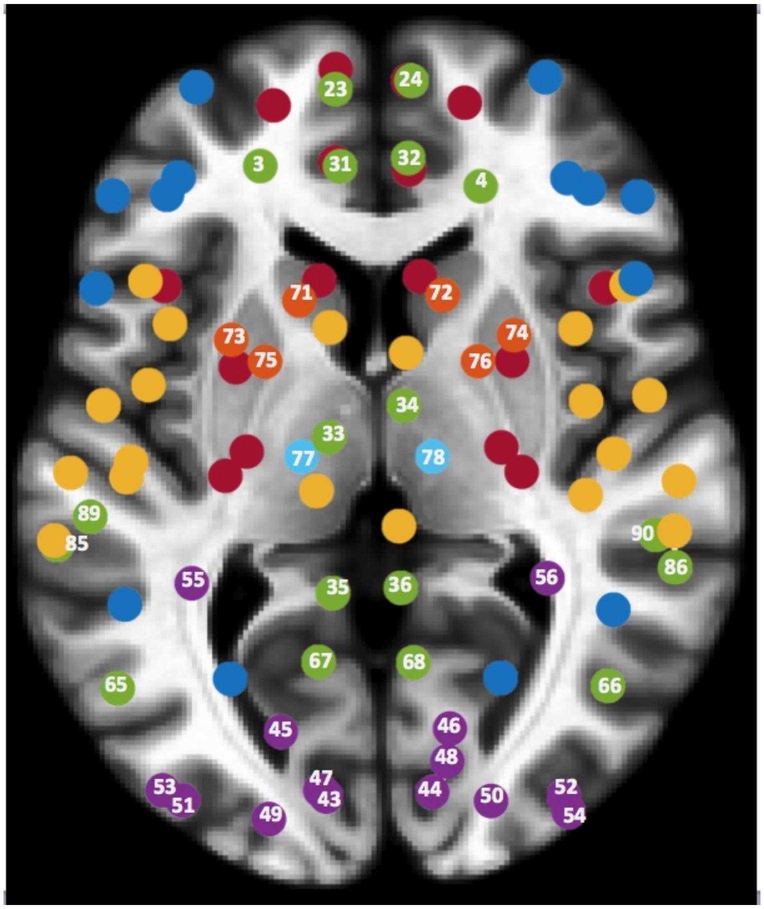

Figure 1: *Brain regions within functional subnetworks (modules): sagittal view.* Color labeling is the same as in Figure 5 in the text, namely: Red = Limbic; blue = Fronto-parietal; orange = Basal-ganglia; yellow = Temporo-parietal; purple = Occipital; green = DMN; light blue = thalamus. Numeric labels refer to the 90 ROIs obtained from [7]

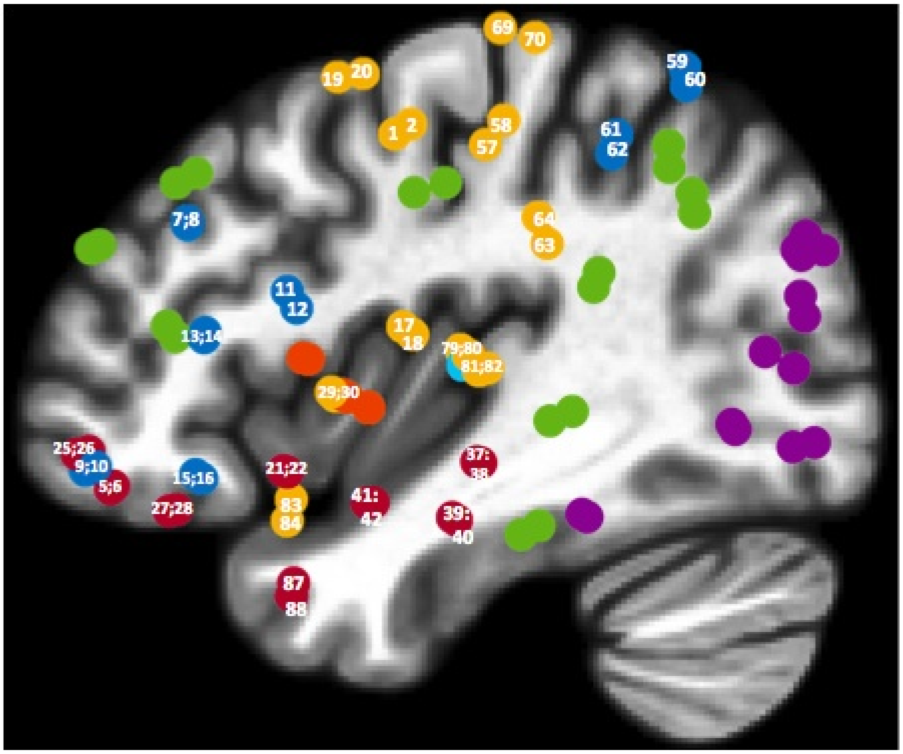

Figure 2: *Brain regions within functional subnetworks (modules), lateral view.* Color labeling is the same as in Figure 5 in the text, namely: Red = Limbic; blue = Fronto-parietal; orange = Basal-ganglia; yellow = Temporo-parietal; purple = Occipital; green = DMN; light blue = thalamus. The numeric labels refer to the 90 ROIs obtained from [7].

3 Overview of the research strategy used in the paper.

3.1 Reckoning the parameters of an Adjacency Matrix.

In Figure 3 a simple network is reported (left panel), together with the corresponding Adjacency Matrix (right panel).

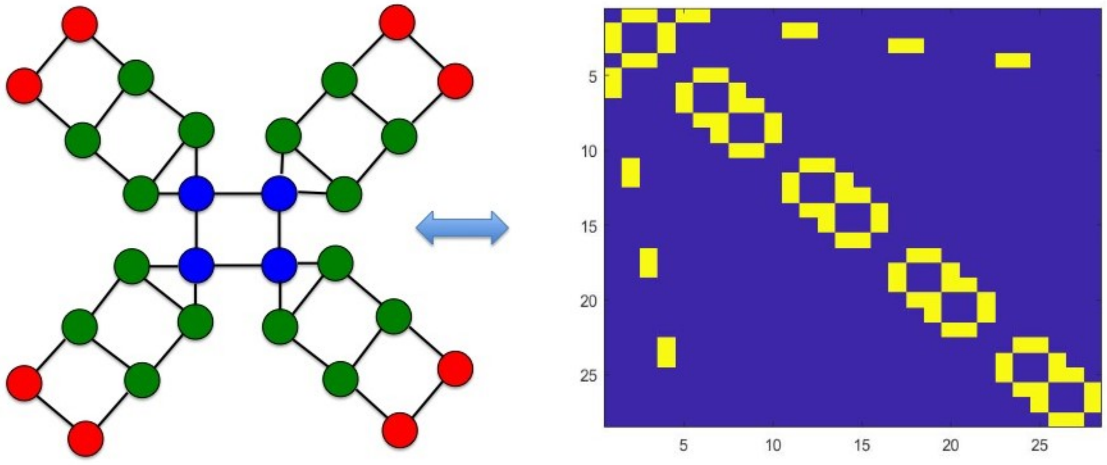

Figure 3: *Matrix and network example.* Left: a network is shown having nodes with node degree = 2, 3 and 4 in red, green and blue, respectively. Right: the corresponding adjacency matrix used by the algorithm to calculate the network indexes.The total number of links is 40 corresponding, roughly, to 10% of all possible connections.

The following sequence of operations, coded in the form of a MATLAB script, can be used to estimate the network parameters, namely the Rich-club, the Local-club and the Feeder-club indexes.

- Count the total amount of links in the network: 40 links, and saved as E_t.
- Find nodes having node degree > 2 (green and blue): 20 nodes, and count the number of links between them: 28 links;
- Find nodes having node degree > 3 (blue): 4, and count the number of links between them: 4;

- Find nodes having node degree > 4: 0;
- The number of nodes and of links are saved as N_{rich} and E_{rich}, respectively.
- Find nodes having node degree $\leq$ 2 (red): 8, and count the number of links between them: 4;
- Find nodes having node degree $\leq$ 3 (red and green): 24, and count the number of links between them: 28;
- Find nodes having node degree $\leq$ 4 (red, green and blue): 28, and counts the number of links: 40;
- The number of nodes and of links are saved as N_{local} and E_{local}, respectively.
- Reckon the number of links among nodes with node degree >2 (green and blue) and $\leq$2 (red) –> E_t - (E_{rich}+ E_{local}): 40 - (28+4)= 8;
- Reckon the number of links among nodes with node degree > 3 (blue) and $\leq$ 3 (red and green) –> 40 - (4 + 28)= 8;
- Reckon the number of links among nodes with node degree > 4 (zero) and $\leq$3 (red, green and blue): 0;
- The number of links are saved as E_{feeder}.
- Reckon the rich-club coefficient (Rc): $2 * E_{rich}$ / [N_{rich}*(N_{rich}-1)];
- Reckon the local-club coefficient (Lc): $2 * E_{local}$ / [N_{local}*(N_{local}-1];
- Reckon the feeder-club coefficient (Fc): E_{feeder} / (N_{rich}*N_{local}).

The values generated by the previous sequence of operations on the basis of the network and of the Adjacency Matrix reported In Figure 3 are contained in Table 1. The MATLAB function able to generate the values is the following:

[Rc Lc Fc Erich Elocal Efeeder Nrich Nlocal] = club(R,2,4)

where R is the 28 x 28 matrix of the network in the example and 2 and 4 are the minimum and the maximum node degree values in the network. The corresponding MATLAB script can be obtained from one of the authors (F.P.) upon request.

**node degree**	N_{rich}	N_{local}	E_{rich}	E_{local}	E_{feeder}	**Rc**	**Lc**	**Fc**
2	20	8	28	4	8	0.15	0.14	0.05
3	4	24	4	28	8	0.67	0.10	0.08
4	0	28	0	40	0	0	0.11	0

Table 1: **Network Parameters from the Adjacency Matrix in Figure 3.**

3.2 Schematics of the analytical steps used in this paper.

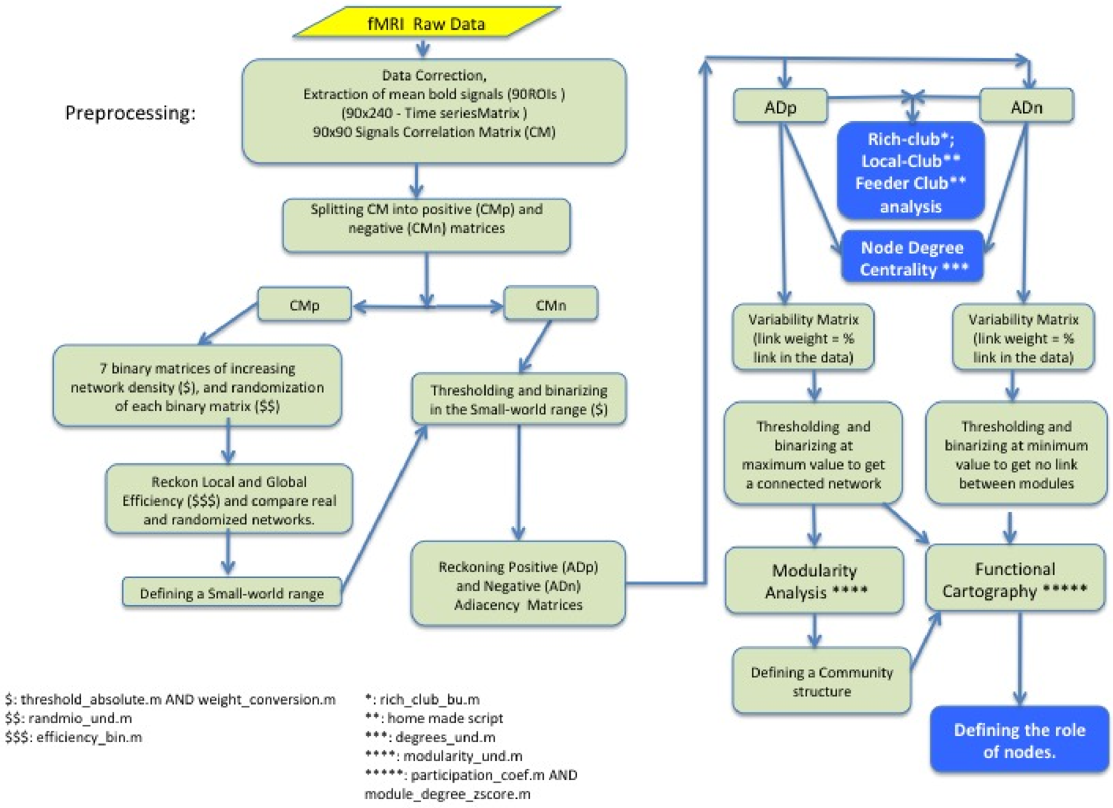

Figure 4: *Flow Chart of the computing strategy followed in this paper.* The scripts indicated by asterisks or ($) in the flow-chart have been taken from `https://sites.google.com/site/bctnet/`. The original script named 'home made' is reported below.

References

[1] M. van den Heuvel and O. Sporns. Rich-club organization of the human connectome. *J Neurosci.*, 31(44):15775–15786, 2011. 1

[2] V. Latora and M. Marchiori. Efficient behavior of small-world networks. *Phys. Rev. Lett.*, 87, 2001. 2

[3] Rubinov M. and Sporns O. Weight-conserving characterization of complex functional brain networks. *Neuroimage*, 56:2068–2079, 2011. 2

[4] M.E.J. Newman. Finding community structure in networks using the eigenvectors of matrices. *Phys. Rev. E*, 74:036104, 2006. 2

[5] L.A.N. Guimera, R. Amaral. Cartography of complex networks: Modules and universal roles. *J Stat Mech Theor Exp.*, page 2005P02001, 2005. 3

[6] S. Maslov and K. Sneppen. Specificity and stability in topology of protein networks. *Science*, 296(6):910–3, 2002. 3

[7] N. Tzourio-Mazoyer, B. Landeau, D. Papathanassiou, F. Crivello, O. Etard, N. Delcroix, B. Mazoyer, and M. Joliot. Automated anatomical labeling of activations in spm using a macroscopic anatomical parcellation of the mni mri single-subject brain. *Neuroimage*, 15(1):273–89, 2002. 4, 5
